# Impact of Rhythm vs. Rate Control in Atrial Fibrillation on the Long-Term Outcome of Patients Undergoing Transcatheter Edge-to-Edge Mitral Valve Repair

**DOI:** 10.3390/jcm10215044

**Published:** 2021-10-28

**Authors:** Christian Waechter, Felix Ausbuettel, Georgios Chatzis, Juan Cheko, Dieter Fischer, Holger Nef, Sebastian Barth, Philipp Halbfass, Thomas Deneke, Sebastian Kerber, Jan Kikec, Hans-Helge Mueller, Dimitar Divchev, Bernhard Schieffer, Ulrich Luesebrink

**Affiliations:** 1 Department of Cardiology, University Hospital Marburg, Baldingerstraße, 35043 Marburg, Germany; christian.waechter@staff.uni-marburg.de (C.W.); felix@ausbuettel.info (F.A.); hatzisgeorgios@gmail.com (G.C.); juanacheko@yahoo.de (J.C.); dimitar.divchev@uk-gm.de (D.D.); bernhard.schieffer@med.uni-marburg.de (B.S.); 2 Department of Cardiology, Cardiovascular Center Rotenburg/Fulda, Heinz-Meise-Straße 100, 36199 Rotenburg/Fulda, Germany; dieterfischer@yahoo.de (D.F.); holger.nef@me.com (H.N.); 3 Department of Cardiology, University Hospital Giessen, Klinikstraße 33, 35392 Giessen, Germany; 4 Department of Cardiology, Cardiovascular Center Bad Neustadt/Saale, Von-Guttenberg-Straße 11, 97616 Bad Neustadt/Saale, Germany; sebastian.barth@campus-nes.de (S.B.); halbfass@gmx.net (P.H.); thomas.deneke@campus-nes.de (T.D.); sebastian.kerber@campus-nes.de (S.K.); jan.kikec@campus-nes.de (J.K.); 5 Institute for Bioinformatics and Biostatistics, Philipps University, Bunsenstraße 3, 35037 Marburg, Germany; hans-helge.mueller@staff.uni-marburg.de

**Keywords:** MitraClip, rhythm-control, rate-control, mitral regurgitation, heart failure, pharmacological rhythm control, elderly

## Abstract

Atrial fibrillation (AF) is a highly prevalent comorbidity in patients with severe mitral valve regurgitation (MR) undergoing transcatheter mitral valve repair (TMVR) and has been shown to significantly worsen their outcome. However, data on the impact of AF treatment strategy in this rapidly growing cohort of patients is unknown. In a multicenter, observational cohort study, 542 consecutive patients undergoing TMVR were enrolled, and subsequently, comprehensive survival analyses according to AF status and therapy were performed using propensity score matching and Cox regression. In the analyzed cohort, 373 (73.3%) of the TMVR patients had concomitant AF. Of these patients, 212 (59%) were on rate control therapy and 161 (41%) were on rhythm control therapy. At 3 years, significantly reduced cumulative survival was observed for patients on rhythm compared to patients on rate control (46.7% (75/161) vs. 56.5% (91/161), *p* = 0.032). Amiodarone was used to a substantial extent for rhythm control and found to be an independent mortality predictor (Hazard Ratio 1.5, 95%CI 1.1–2.1, *p* = 0.04). The adverse outcome of concomitant AF in TMVR patients was confirmed (AF: 47.3% (126/266) vs. non-AF: 58.3% (78/133), *p* = 0.047). Rhythm control achieved almost exclusively pharmacologically is associated with an adverse outcome compared to the rate control of AF in TMVR. This raises awareness of the importance of AF and its treatment, as this seems to be a promising key point for improving the prognosis of TMVR patients.

## 1. Introduction

With a prevalence of nearly 10% in the population over 75 years of age, mitral regurgitation (MR) is one of the most common valvular heart diseases in industrialized countries [1]. However, especially in this elderly population of patients, the oftentimes high degree of advanced heart failure and other comorbidities often prohibit the prognostically favorable surgical repair due to unacceptably high perioperative risk. For this specific cohort, transcatheter edge-to-edge repair with the MitraClip^®^ device (Abbott Vascular, Santa Clara, CA, USA) has proven to be an excellent treatment option, as it has been shown to be safe and effective in alleviating heart failure symptoms [2] and even mortality [3] in different MR etiologies. With a prevalence of up to 73.3% in real-world data, atrial fibrillation (AF) is exceedingly frequent and inseparably linked to MR [4]. This emphasizes in particular the complex pathophysiological interactions of these two important diseases. In this context, a plethora of recent research show that patients undergoing transcatheter mitral valve repair (TMVR) with concomitant AF have a significantly increased mortality in the medium and long term [5,6,7,8]. This highlights why AF cannot be considered as a negligible benign concomitant disease and why additional strategies are urgently needed to improve the prognosis of this special and still growing cohort of patients. While there is strong evidence for prognostically favorable concomitant rhythm control of AF in the collective of surgically treated MR patients, reflected in a class I recommendation in the relevant guidelines, data on the effects of therapy for AF in the TMVR cohort are lacking [9,10,11]. Therefore, the aim of the present study is to elucidate the impact of different strategies for the treatment of concomitant AF on the outcome of TMVR patients.

## 2. Methods

### 2.1. Data Collection and Definitions

Data from all consecutive patients scheduled for percutaneous therapy of MR using the MitraClip^®^ device in three tertiary heart centers in Germany between October 2011 and May 2020 were collected in registries in each hospital and subsequently pooled for analysis. Details on patient selection, procedural aspects as well as details on definitions of AF types and therapies have recently been published [4,12]. In brief, the definition of AF types has been made in accordance with the guidelines of the European Society of Cardiology [13]. Hence, paroxysmal AF was defined as AF lasting a maximum of seven days. Accordingly, persistent AF was defined as AF lasting longer than seven days with or without cardioversion, but still aspiring to a rhythm control strategy. All AF episodes that lasted longer than or were terminated after seven days were defined as persistent AF. Permanent AF was defined if no rhythm control interventions were pursued anymore. Furthermore, all studied patients with paroxysmal AF were defined to be treated with the objective of rhythm control. Persistent AF patients were defined to be on rate control if the medication consists of beta blocker only, a combination of beta blocker and digitalis or if a pacemaker has been implanted with or without an additional AV node ablation. Rhythm control was assumed to be intended if patients with persistent AF were treated with class I or class III antiarrhythmic drugs with or without beta blocker or if pulmonary vein isolation was performed up to 24 months prior to TMVR. The indication for amiodarone for rhythm control of AF was based on the corresponding valid guidelines of the European or German cardiac societies. According to these guidelines, except for class II and class III antiarrhythmics, all other antiarrhythmics are not recommended or even contraindicated in the presence of relevant structural heart diseases, which was the case in the majority of the patients studied. Amiodarone was not used when rate control could not be achieved with other negative dromotropic drugs. Other indications for amiodarone, such as treatment of coexisting ventricular arrhythmias, were considered separately and accounted for accordingly. Amiodarone was used orally only, and the dosing was left to the discretion of the treating physician. However, it can be stated that a daily dose of 200 mg is considered standard in the participating centers.

Furthermore, the indication for pacemaker therapy was carefully verified in each case and only included in the analysis if the indication was clearly related to rate control therapy in concomitant AF. Major adverse cardiac and cerebrovascular events (MACCE) were defined as the occurrence of a cerebral and/or systemic thromboembolic event, a hemorrhage requiring intervention and/or transfusion or in-hospital death from a cardiovascular cause. The local ethics committee approved the study.

### 2.2. Statistical Analysis

All statistical analyses were performed by using R Studio V3.6.1 (R Foundation for Statistical Computing, Vienna, Austria), including the “MatchIt”, “survival”, “survminer”, “dplyr”, “stddiff” and “My.Stepwise” Packages as well as GraphPad Prism 6.0 (GraphPad Software, La Jolla, CA, USA). Regarding categorical variables, data are presented as frequencies and percentages (%), continuous variables are presented with mean and standard deviation for the standard distributed variables and with median and interquartile ranges (IQR; 25th–75th percentile) as well as first and third quartile (Q1; Q3) for non-standard distributed variables. A two-sided *p*-value of <0.05 was considered statistically significant. Differences between two groups were compared by using a Chi-squared test and Fisher’s exact test for categorial variables, student’s *t*-test for standard distributed variables and Wilcoxon rank sum-test for non-standard distributed variables. In order to be able to consider differences in the baseline characteristics and achieve consequentially an unbiased comparison between both AF and non-AF patient’s outcomes and patients on rhythm and rate control, a propensity score matching analysis was performed using the nearest neighbor matching with a caliper width set at 10% standardized difference of the logit of the estimated propensity scores. To include as many patients as possible from the collective examined for the analysis, a 2:1 matching was carried out for the long-term survival comparison of AF and non-AF patients. The matching ratio was based on the group with the lowest number of patients. Analogously, the long-term survival of AF patients on rhythm and in rate control was compared using a 1:1 ratio of propensity score matching (PSM). With regard to appropriate matching parameters, significantly different parameters in the corresponding baseline characteristics and already published and generally accepted mortality predictors as well as mortality predictors were used, which were revealed or confirmed by uni- and multivariable Cox regression analysis in the present collective. The selected matching parameters were male sex, chronic obstructive pulmonary disease (COPD), coronary artery disease, prior stroke, diabetes mellitus, pre-existing implantable cardioverter/defibrillator, advanced age (>75 years), values > 10% in the STS-Risk-Score, glomerular filtration rate (GFR), left ventricular function as well as concomitant high grade tricuspid valve regurgitation (TR). After matching, the time-to-event analysis for the different AF types and treatment strategies were carried out by using the Kaplan–Meier method, while differences between groups were compared with the Log-rank test. Univariable as well as multivariable Cox regression was used to determine independent predictors of mortality.

### 2.3. Missing Data

In case the follow-up data were insufficient, it was supplemented by survival query to the registry office for patients who were lost to follow-up. Despite the efforts made, 18 patients (3,5%) were lost to follow-up during the indicated study period due to an uncommunicated change of residence. However, there were no indications of an informative missing and of a significant impact of the “lost to follow-up” patients on the presented results.

### 2.4. Results

In the participating tertiary heart centers, 542 consecutive patients with severe MR scheduled for edge-to-edge TMVR with the use of the MitraClip^®^ device were identified. Thirty-six patients (6.6%) were excluded from further analysis due to an unsuccessful procedure. As previously reported, 373 (73.3%) patients of the analyzed cohort suffered from coexistent AF, with the presence of AF not correlating with the success of the TMVR procedure (Odds Ratio (OR) 0.62, 95%-CI, 0.29–1.39, *p* = 0.27) [4]. Apart from this, the incidence of MACCE and the in-hospital death from any cause did not differ significantly in AF and non-AF patients (15/373 (3.5%) vs. 7/133 (5.3%), *p* = 0.4; 15/373 (4.0%) vs. 6/133 (4.5%), *p* = 0.8). The median follow-up period of the analyzed collective was 475 days (IQR 555 days).

## 3. Long-Term Outcome of AF and Non-AF Patients

**Figure 1:**
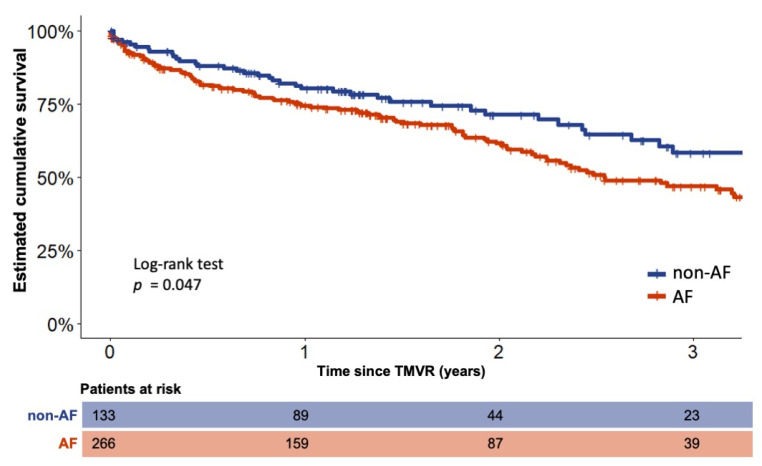
Estimated cumulative survival of patients with and without concomitant AF Kaplan–Meier plots of patients with and without a history of concomitant atrial fibrillation (AF) after propensity score matching (PSM). TMVR—transcatheter mitral valve repair.

Within this reported cohort, the 133 patients without a history of AF were compared with AF patients for survival using propensity score matching (PSM) with a ratio of 1:2. A significantly reduced estimated cumulative survival of AF compared to non-AF patients was observed after three years (126/266 (47.3%) vs. 78/133 (58.3%), HR 1.4, 95%-CI 1.004–2.03, *p* = 0.047). The related Kaplan–Meier plot is shown in Figure 1. Post-PSM multivariable Cox regression revealed severe tricuspid regurgitation (Hazard Ratio (HR) 1.2, 95%-CI 1.05–1.5, *p* = 0.008) and male sex (HR 1.7, 95%-CI 1.2–2.3, *p* = 0.001) as significant negative predictors of long-term survival. An influence of MACCE related to the initial procedure as well as in-hospital mortality can be excluded, because there were no statistically significant differences between the groups in this respect. Neither a propensity score-matched Kaplan–Meier analysis, nor multivariable Cox regression analysis revealed an influence of MR etiology on outcome when comparing AF and non-AF patients. The baseline characteristics as well as the distribution of the heart failure and antiarrhythmic medication of AF and non-AF patients before and after PSM are displayed in Table 1.

## 4. Long-Term Outcome of Different AF Types and AF Treatment Strategies

With regard to the impact of the different AF entities, such as permanent and non-permanent AF, no statistically significant differences in the cumulative survival after 3 years could be detected even after PSM (69/152 (45.5%) vs. 75/152 (49.4%), *p* = 0.91). The corresponding data on patient characteristics and the visualization of the Kaplan–Meier survival function are attached as Table S1 and Figure S1, respectively.

**Figure 2:**
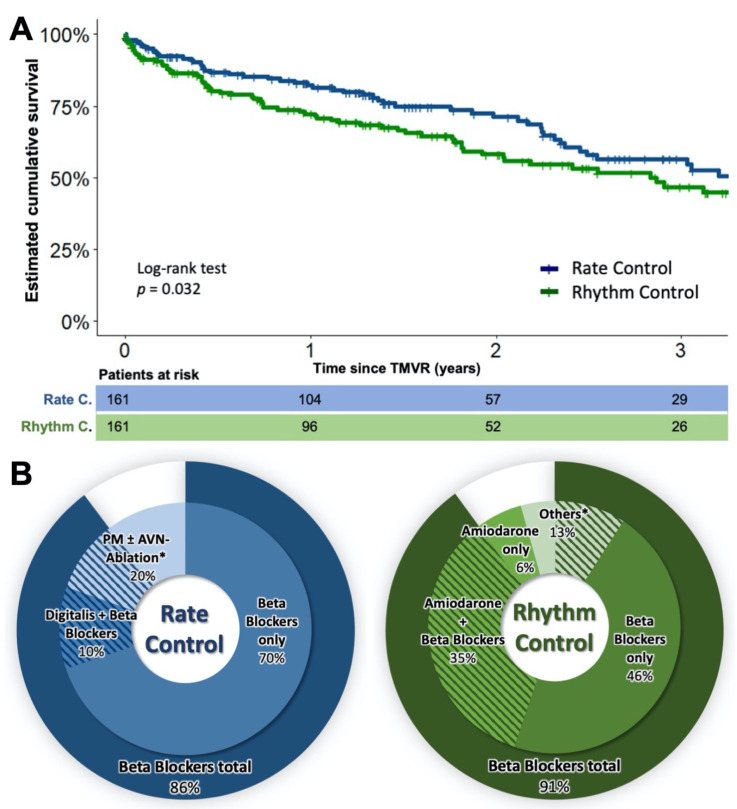
Estimated cumulative survival of patients under rate and rhythm control therapy (**A**) and AF-related therapies (**B**). (**A**) Kaplan–Meier plots of patients under rate and under rhythm control therapy after PSM. (**B**) Overview of AF-specific therapies. The left diagram indicates the distribution of rate control therapies. The right diagram indicates the distribution of rhythm control therapies. The asterisks highlight the sub-groups in which beta-blockers were also used in some cases. Here, the shaded area indicates the proportion of beta-blockers in the respective sub-group. TMVR—transcatheter mitral valve repair. PM—pacemaker therapy. AVN—AV node.

Comparing the outcomes of the underlying AF treatment strategy after 1:1 PSM, a significantly reduced cumulative survival of the rhythm-controlled compared to the rate-controlled patients was observed after three years (75/161 (46.7%) vs. 91/161 (56.5%), HR 1.5, 95%-CI 1.03–2.06, *p* = 0.032). The baseline characteristics of the two groups before and after PSM are shown in Table 2 and the related Kaplan–Meier plot is presented in Figure 2, panel A. Cox regression analyses performed within groups adequately balanced by PSM revealed male sex is a significant negative predictor of long-term survival in multivariable regression (HR 1.5, 95%-CI 1.02–2.1, *p* = 0.04). Accordingly, the usage of amiodarone also emerged as a significant negative predictor of long-term survival (HR 1.5; 95%-CI 1.1–2.1, *p* = 0.04) in univariable regression. The use of Digitalis did not prove to be a significant predictor of mortality either, not in the multivariable nor in the univariable Cox regression leading to the fact that effects of differences in this baseline characteristic persisting after PSM is very unlikely. Analogous to the previous section, neither propensity score-matched Kaplan–Meier analysis nor multivariable Cox regression analysis showed an influence of MR etiology on outcome when comparing atrial fibrillation therapy strategies. Figure 2, panel B provides a detailed overview of the distribution of the different AF-specific therapies in terms of rhythm control and rate control.

## 5. Use of Amiodarone within the Entire Cohort and Overlapping Indications

To further elucidate the role of amiodarone independent of treatment strategy in AF, we first reviewed amiodarone use in the entire cohort (see Table 1). Additionally, in the overall population studied, the use of amiodarone predicted mortality (HR 1.5, 95%-CI 1.1–2.1, *p* = 0.02) after univariable Cox regression. Subsequently, the different indications for amiodarone use were disaggregated and investigated. In the non-AF cohort amiodarone was used in 8 patients (6%) for the treatment of ventricular tachycardia (VT). In the entire AF cohort, amiodarone was used in 84 patients (22.5%): of these, 72 patients used amiodarone for rhythm control in AF and the remaining 12 patients used amiodaron also for partial concomitant treatment of VT. Of the latter, 8 patients were in the rate control group and 4 patients in the rhythm control group. Since amiodarone also exerts a rhythm-controlling effect with respect to atrial fibrillation that cannot be sharply separated or excluded clinically, analyses were performed with and without these patients with overlapping indications for amiodarone. Even after exclusion of patients with concomitant VT, the statistically significant negative effect of amiodarone treatment on mortality persists (HR 1.5, 95%-CI 1.02–2.06, *p* = 0.047). Likewise, the propensity score-adjusted treatment cohort, excluding patients with concomitant VT, also shows a statistically significant worsening of overall survival in the rhythm control group (see Figure S2).

## 6. Discussion

This analysis, for the first time, provides detailed data on the long-term clinical outcome of TMVR patients under special consideration of coexistent atrial fibrillation and its underlying treatment regimen in a well-characterized large multicenter cohort of more than 500 patients.

The following results can be highlighted synoptically: (i) the concomitance of AF did not affect the procedural success, the rate of MACCE or the in hospital-mortality, but (ii) is significantly associated with a worse long-term clinical outcome compared to sinus rhythm; (iii) patients with permanent or non-permanent AF subtypes did not differ in the long-term clinical outcomes, but the underlying AF treatment regime seems to have a substantial impact on this; (iv) pharmacologically rhythm-controlled patients exhibited a significantly adverse long-term clinical outcome compared to rate-controlled patients.

According to current data from published trials and registries, our investigated population, at 73.3%, exhibits the highest prevalence of concomitant AF in a TMVR cohort. Underlining the otherwise comparability of the studied cohort, largely similar results were achieved regarding MR etiology, procedural characteristics, technical success, MR reduction and periprocedural MACCE. The already growing evidence of an adverse outcome of AF compared to non-AF patients, as reported for example by the groups of Velu, Keßler and Arora or by two recent meta-analyzes, were, thus, confirmed [5,6,8,14,15]. This, again, highlights the urgent need for additional strategies to improve the prognosis of this important and further growing cohort of patients. Treading this path, we have explored the effects of AF type, AF treatment and concomitant influencing factors using complex statistical models. While in the present study, the subtype of AF had no significant impact on the long-term survival, a significantly worse outcome can be demonstrated for patients on rhythm control compared to patients on rate control. Thus, our results contradict those available in collectives of surgically treated patients. As a representative example, the recent results of Grigioni and colleagues can be cited [16]. According to these, the long-term outcome in surgically treated patients with concomitant paroxysmal AF is significantly less favorable and worsens with increasing AF burden. Furthermore, the evidence for positive effects on mortality by simultaneous surgical ablation of AF during mitral valve surgery mentioned in the introduction opposes the present results [9]. If our results are interpreted in comparison with AF patients without concomitant relevant MR, they may also seem unexpected or even controversial in light of recent findings on the positive prognostic effects of rhythm control therapy, as for example reported in the “Catheter Ablation versus Standard Conventional Therapy in Patients with Left Ventricular Dysfunction and Atrial Fibrillation” (CASTLE-AF) trial and in recent pooled analyses of randomized data or meta-analyses [17,18,19]. However, the rhythm control strategies, which are currently at the center of the debate, mainly focus on interventional rhythm control through catheter ablation, whereas in the present study, rhythm control was almost exclusively pharmacological. The above cited patient collectives analyzed to compare rate and rhythm control are significantly younger, have less advanced heart failure, fewer comorbidities, and apparently do not have severe MR, which would be an indication for surgical valve repair ideally combined with surgical AF treatment. Even the CASTLE-AF collective, which is the reported collective with the most advanced heart failure to date, has a 14-year lower median age and performs a NYHA class better than the present TMVR collective, with the majority of the patients being studied in NYHA functional class III [17]. In this context, the results of the recently published subgroup analysis of the CASTLE-AF trial, showing no significant differences in the long-term prognosis of pharmacological rhythm- vs. rate-controlled patients, are only comparable to a limited extent [20]. These differences in patient characteristics, some of which are fundamental, are probably the most likely reason for the contradictory result of our study compared with the available data obtained from surgically treated patients or from AF patients without relevant MR. This highlights the uniqueness of the TMVR collectives. Indeed, when comparing the two AF therapy strategies in patient collectives that were much more similar to the present collective in terms of age and the degree of comorbidity, a trend toward the inferiority of rhythm control therapy was evident. Due to the lack of more recent robust data from randomized controlled trials (RCT) addressing these patients, data from the “Atrial Fibrillation and Congestive Heart Failure” (AF-CHF) and the “Atrial Fibrillation Follow-up Investigation of Rhythm Management” (AFFIRM) trials so far provide guidance [21,22]. These RCTs did not show a survival advantage of pharmacological rhythm control or even showed an excess mortality of rhythm- compared to rate-controlled patients. Along these lines, a corresponding subgroup analysis of the AFFIRM data from septuagenarians showed a significantly higher mortality of patients treated with rhythm- compared to rate-control [23]. Additionally, a recently published analysis of this study group stratified by the modified version of the Charlson Comorbidity Index (mCCI) shows a similar trend towards a worse outcome of patients with high mCCI under medical rhythm control, which just failed to reach statistical significance due to being underpowered [24]. In these collectives with almost exclusively drug-based rhythm control, it is assumed that adverse side effects of the antiarrhythmics contribute significantly to the poorer prognosis of the rhythm-controlled patients. Based on results of the “Cardiac Arrhythmia Suppression Trial” (CAST) and further analyzes, deleterious effects of class I antiarrhythmics were mainly taken into account for these findings, but the use of amiodarone also seems to be problematic [25]. Class I antiarrhythmics were only of negligible use without any influence on the present TMVR patients. In addition, other rhythm-specific medications, or interventions such as the use of beta blockers, pacemakers with or without additional AV node ablation or pulmonary vein isolation, were not shown to be predictors of mortality. In contrast, amiodarone was used to a substantial extent for rhythm control in the present collective. Additionally, a statistically significant association between amiodarone use and mortality was demonstrated by accepted regression analysis in a collective that was appropriately balanced by PSM. With all caution in interpreting this result, especially given the observational study design, it may nevertheless provide clues to explain the poorer outcome of the rhythm control patients. Accordingly, a very recent study demonstrates an association of amiodarone use in elderly patients with an increased short-term mortality when hospitalized due to AF [26]. Due to the multimorbidity and polypharmacy, elderly patients, especially those with coexisting heart failure, as in the present collective, show a particular high risk of being affected by adverse side effects and toxicities related to the use of amiodarone. However, since our study did not aim to assess the potential side effects or toxicities associated with amiodarone use, we can only speculate about causal relationships at this point.

## 7. Limitations

Due to the nature of an observational cohort study, the results cannot prove a causal relationship, and despite carefully adjusting baseline differences, a possibility of residual confounding remains. Compared with clinical trials, the proportion of missing data, albeit small, and the setting of a registry may limit accuracy, which may limit internal validity. Furthermore, we cannot assess the effectiveness of the AF therapies regarding stability of sinus rhythm, as relevant data were not fully recorded. Nonetheless, highly relevant clinical endpoints were addressed.

## 8. Conclusions

Heart failure and AF represent a highly prevalent combination of morbidities in real-world TMVR patients and are associated with a very poor long-term prognosis. Here, we demonstrate that the treatment strategy of concomitant AF has a significant impact on the outcome of TMVR patients. Hence, it can be concluded that pharmacological rhythm control is associated with a worse outcome compared to rate control. In this context, amiodarone was used to a substantial extent for rhythm control and its use was found to be associated with an increase in mortality. With the right amount of caution, this could be interpreted as an indication that the use of amiodarone should be carefully considered and vigilantly monitored. Certainly, the present study, on the one hand, raises awareness of the importance of the treatment of concomitant AF, as this seems to be a promising key point for improving the prognosis of TMVR patients. On the other hand, it highlights the vulnerability and uniqueness of these patients. Thus, contemporary strategies for rhythm control, which have been shown to be highly effective and safe in related patient populations and are associated with positive prognostic effects, need to be investigated in the growing cohort of TMVR patients.

## Figures and Tables

**Table 1:** Baseline characteristics of patients with and without concomitant AF before and after propensity score matching.

		Before Propensity Score Matching			After Propensity Score Matching		
	Total *n* = 506	Non-AF *n* = 133	AF *n* = 373	*p*-Value	Non-AF *n* = 133	AF *n* = 266	*p*-Value
Age (years)	78.1 ± 7.8	77.4 ± 9.1	78.4 ± 7.3	0.3	77.4 ± 9.1	77.8 ± 7.3	0.6
Male sex	62.9%	60.9%	63.5%	0.6	60.9%	63.2%	0.6
EuroSCORE II (Q1; Q3)	19.97% (10.7; 33.6)	19.11% (12.1; 34.0)	19.98% (4.7; 13.6)	0.7	19.1% (12.1; 34.0)	20.0% (10.7; 33.4)	1
STS-Risk-Score (Q1; Q3)	7.4% (4.7; 13.4)	7.3% (4.2; 12.8)	7.6% (4.7; 13.6)	0.6	7.3% (4.2; 12.8)	7.7% (4.8; 15.2)	0.3
NYHA class I NYHA class II NYHA class III NYHA class IV	0.2% 4.7% 71.0% 24.1%	0% 5.3% 73.7% 21.0%	0.3% 4.6% 70.0% 25.2%	0.7	0.0% 5.3% 73.7% 21.1%	0.4% 2.6% 69.2% 27.8%	0.2
COPD	20.6%	22.6%	19.8%	0.5	22.6%	21.1%	0.7
Coronary artery disease	67.0%	77.4%	63.3%	0.002	77.4%	77.1%	0.9
Prior CABG surgery	26.7%	35.3%	23.6%	0.008	35.3%	28.2%	0.2
Prior PCI	55.1%	62.4%	52.5%	0.049	62.4%	60.2%	0.7
Diabetes mellitus	33.4%	35.3%	32.7%	0.6	35.3%	35.7%	0.9
Art. Hypertension	80.0%	79.7%	80.2%	0.9	79.7%	79.7%	1
Prior Stroke	10.9%	11.3%	10.7%	0.9	11.3%	9.8%	0.7
Pre-existing ICD	26.9%	26.3%	27.1%	0.9	26.3%	29.7%	0.5
Pre-existing CRT	11.7%	11.3%	11.8%	0.9	11.3%	13.5%	0.5
GFR > 60 mL/Min GFR 30–59 mL/Min GFR < 30 mL/Min	25.5% 55.3% 19.0%	30.1% 56.6% 19.3%	23.9% 51.9% 18%	0.5	30.1% 51.9% 18%	25.9% 55.6% 18.4%	0.7
NT-pro BNP (ng/L) (Q1; Q3)	2945 (1089; 5989)	2960 (1129; 7000)	2935 (1085; 5671)	0.4	2960 (1129; 7000)	3031 (1056; 5994)	0.5
LV function > 45% LV function 30–44% LV function < 30%	38.7% 34.6% 26.7%	30.1% 38.3% 31.6%	41.8% 33.2% 24.9%	0.06	30.1% 38.3% 31.6%	38% 35% 27.1%	0.3
TR grade III	18.8%	11.3%	21.4%	0.01	11.3%	10.5%	0.8
Degenerative MR etiology Functional MR etiology Combined MR etiology	27.5% 64.6% 7.9%	27.1% 65.4% 7.5%	27.6% 64.3% 8.0%	0.9	27.1% 65.4% 7.5%	25.6% 66.9% 7.5%	0.9
**Procedural characteristics**							
Mean procedure duration (min)	108.2 ± 63.1	104.0 ± 53.0	109.6 ± 66.3	0.3	104 ± 53	110 ± 67	0.4
Postinterventional no MR Postinterventional MR grade I Postinterventional MR grade II Postinterventional MR grade III	24.7% 62.9% 12.0% 0.4%	23.3% 62.4% 13.5% 0.8%	25.2% 63.0% 11.5% 0.3%	0.6	23.3% 62.4% 13.5% 0.8%	25.2% 62.4% 12.4% 0.0%	0.6
1 Clip implanted 2 Clips implanted 3 Clips implanted 4 Clips implanted	37.9% 52.8% 9.1% 0.2%	36.8% 51.9% 11.3% 0.0%	36.3% 53.1% 8.3% 0.3%	0.7	36.8% 51.9% 11.3% 0.0%	42.1% 50.4% 7.1% 0.4%	0.4
Length of hospital stay (days) (Q1; Q3)	7 (5; 10)	7 (4; 9)	7 (5; 10)	0.2	7 (4; 9)	7 (5; 10)	0.5
MACCE	4.4%	5.3%	3.5%	0.4	5.3%	4.1%	0.6
In-hospital death from any cause	4.2%	4.5%	4.0%	0.8	4.5%	4.5%	1
**Heart Failure and anti-arrhythmic medication**							
ACE-/AT1 Inhibitors	74.1%	72.9%	74.5%	0.7	72.9%	75.2%	0.6
ARN Inhibitor	7.9%	7.5%	8.0%	0.8	7.5%	7.9%	1
Beta Blockers	87.9%	87.2%	88.2%	0.7	87.2%	90.2%	0.4
Loop diuretics	89.5%	87.2%	90.3%	0.3	87.2%	90.2%	0.4
Thiazid diuretics	21.3%	21.8%	21.1%	0.9	21.8%	19.5%	0.6
Aldosteron antagonists	48.0%	47.4%	48.3%	0.9	47.4%	48.5%	0.9
Ivabradin	1.4%	3.8%	0.5%	0.015	3.8%	0.8%	0.04
Digitalis	7.7%	0.0%	10.5%	<0.0001	0.0%	10.2%	<0.0001
Amiodarone	18.2%	6.0%	22.5%	<0.0001	6.0%	22.9%	<0.0001

Data presented as percentages, mean ± SD or median with first quartile and third quartile 3 (Q1; Q3). AF—atrial fibrillation. NYHA—New York Heart Association. COPD—chronic obstructive pulmonary disease. CABG—coronary artery bypass graft surgery. PCI—percutaneous coronary intervention. ICD—implantable cardioverter defibrillator. CRT—cardiac resynchronization therapy. GFR—glomerular filtration rate. NT-proBNP—N-terminal-pro hormone brain natriuretic peptide. LV function—left ventricular function. TR—tricuspid regurgitation. MR—mitral regurgitation. MACCE—major adverse cardiac and cerebrovascular events. ACE—angiotensin converting enzyme. AT1—angiotensin II type 1 receptor. ARN—angiotensin receptor neprylisin. *p*-values describe differences between patients with and without a history of AF.

**Table 2:** Baseline characteristics of patients under rhythm and rate control therapy before and after propensity score matching.

	Before Propensity Score Matching			After Propensity Score Matching		
	Rhythm-Control *n* = 161	Rate-Control *n* = 212	*p*-Value	Rhythm-Control *n* = 161	Rate-Control *n* = 161	*p*-Value
Age (years)	76.8 ± 8.3	79.5 ± 6.3	<0.0001	76.8 ± 8.3	78.4 ± 6.4	0.06
Male sex	62.1%	64.6%	0.6	62.1%	62.1%	1
EuroSCORE II (Q1; Q3)	20.0% (11.6; 36.2)	19.1% (9.7; 32.5)	0.3	20.0% (11.6; 36.2)	17.5% (9.3; 32.9)	0.2
STS-Risk-Score (Q1; Q3)	7.2% (4.3; 13.0)	7.6% (5.0; 14.3)	0.3	7.2% (4.3; 13.0)	6.8% (4.4; 13.4)	0.9
NYHA class I NYHA class II NYHA class III NYHA class IV	0.0% 5.6% 71.4% 23.0%	0.5% 3.8% 68.9% 26.9%	0.6	0.0% 5.6% 71.4% 23%	0.6% 5% 64.6% 29.8%	0.4
COPD	18.6%	20.8%	0.6	18.6%	18.6%	1
Coronary artery disease	63.4%	63.2%	1	63.4%	64.4%	0.8
Prior CABG surgery	25.5%	22.2%	0.5	25.5%	24.8%	0.9
Prior PCI	55.3%	50.5%	0.4	55.3%	49.1%	0.3
Diabetes mellitus	28.0%	36.3%	0.09	28%	33.5%	0.3
Art. Hypertension	78.9%	81.1%	0.6	78.9%	79.5%	0.9
Prior Stroke	8.1%	12.7%	0.1	8.1%	14.1%	0.1
Pre-existing ICD	32.3%	23.1%	0.05	32.3%	27.6%	0.3
Pre-existing CRT	13.6%	10.4%	0.3	13.7%	9.9%	0.6
GFR > 60 mL/Min GFR 30–59 mL/Min GFR < 30 mL/Min	19.3% 58.4% 21.7%	27.4% 55.2% 17.5%	0.15	19.3% 58.4% 21.7%	28.6% 50.9% 20.5%	0.14
NT-pro BNP (ng/L) (Q1; Q3)	2915 (1055; 5528)	2948 (1109; 5696)	0.7	2915 (1055; 5528)	2935 (1094; 5601)	0.2
LV function > 45% LV function 30–44% LV function < 30%	38.5% 35.4% 26.1%	44.3% 31.6% 24.1%	0.5	38.5% 35.4% 26.1%	44.7% 30.4% 24.8%	0.5
TR grade III	14.3%	26.9%	0.003	14.3%	13.7%	0.9
Degenerative MR etiology Functional MR etiology Combined MR etiology	28.0% 65.2% 6.8%	27.4% 63.7% 9.0%	0.8	28% 65.2% 6.8%	25.5% 65.2% 9.3%	0.7
**Procedural characteristics**						
Mean procedure duration (min)	115.2 ± 68	105.4 ± 64.7	0.2	115 ± 68	106 ± 67	0.2
Postinterventional no MR Postinterventional MR grade I Postinterventional MR grade II Postinterventional MR grade III	24.8% 64.6% 10.6% 0.0%	25.5% 61.8% 12.3% 0.5%	0.9	24.8% 64.6% 10.6% 0.0%	26.1% 60.2% 13.7% 0.0%	0.6
Length of hospital stay (days) (Q1; Q3)	7 (6; 11)	7 (4; 10)	0.3	7 (6; 11)	7 (4; 10)	0.3
MACCE	5.6%	1.9%	0.053	5.6%	1.9%	0.08
In-hospital death from any cause	5.0%	3.3%	0.4	5%	3.7%	0.6
**Heart Failure and anti-arrhythmic medication**						
ACE-/AT1 Inhibitors	76.7%	74.6%	0.6	75.8%	73.9%	0.6
ARN Inhibitor	7.5%	8.6%	0.7	7.5%	8.1%	0.8
Beta Blockers	91.8%	87.1%	0.2	90.7%	85.7%	0.2
Loop diuretics	91.8%	91.4%	0.9	90.7%	88.2%	0.5
Thiazid diuretics	18.2%	23.9%	0.2	18%	24.2%	0.4
Aldosteron antagonists	48.4%	49.3%	0.9	47.8%	47.8%	1
Ivabradin	1.3%	0.0%	0.2	1.2%	0.0%	0.5
Digitalis	4.4%	15.3%	<0.001	4.3%	17.4%	<0.0001
Amiodarone	47.2%	3.8%	<0.0001	47.2%	5.0%	<0.0001
**Oral Anticoagulation**	92.5%	90.1%	0.4	92.5%	89.4%	0.3

Data presented as percentages, mean ± SD or median with first quartile and third quartile (Q1; Q3). AF—atrial fibrillation. NYHA—New York Heart Association. COPD—chronic obstructive pulmonary disease. CABG—coronary artery bypass graft surgery. PCI—percutaneous coronary intervention. ICD—implantable cardioverter defibrillator. CRT—cardiac resynchronization therapy. GFR—glomerular filtration rate. NT-proBNP—N-terminal-pro hormone brain natriuretic peptide. LV function—left ventricular function. TR—tricuspid regurgitation. MR—mitral regurgitation. MACCE—major adverse cardiac and cerebrovascular events. ACE—angiotensin converting enzyme. AT1—angiotensin II type 1 receptor. ARN—angiotensin receptor neprylisin. *p*-values describe differences between patients under rate control therapy or under rhythm control therapy.

## Data Availability

The data presented in this study are available upon request from the corresponding author. The data are not publicly available due to ethical restrictions.

## Supplementary Materials

The following are available online at https://www.mdpi.com/article/10.3390/jcm10215044/s1, Table S1: Baseline characteristics of patients with permanent and non-permanent AF before and after propensity score matching, Figure S1: Estimated cumulative survival of patients with permanent and non-permanent AF, Figure S2: Estimated cumulative survival of patients under rate and rhythm control therapy after exclusion of patients with concomitant ventricular tachycardia.
