# Supplementary material for: Impact of Rhythm vs. Rate Control in Atrial Fibrillation on the Long-Term Outcome of Patients Undergoing Transcatheter Edge-to-Edge Mitral Valve Repair
Source: J Clin Med. 2021 Oct 28;10(21):5044. doi: 10.3390/jcm10215044 (PMC8584691; doi:10.3390/jcm10215044)
Supplement: Supplementary file 1 [file jcm-10-05044-s001.zip › jcm-1402350-supplementary.pdf]

Table S1. Baseline characteristics of patients with permanent and non-permanent AF before and after propensity score matching.

	**before propensity score matching**			**after propensity score matching**		
	**non-permanent AF** ***n*** **= 221**	**permanent AF** ***n*** **= 152**	***p*-value**	**non-permanent AF** *n* = 152	**permanent AF** *n* = 152	***p*-value**
Age (years)	77.9 ± 7.6	79.1 ± 6.9	0.1	77.5 ± 6.6	79.1 ± 6.9	0.1
Male sex	58.4%	71.1%	0.04	66.4%	71.1%	0.5
EuroSCORE II (Q1; Q3)	19.6% (9.4-32.7)	20.1% (14.2; 37)	0.6	20% (9.47; 23.64)	20.1% (14.2; 37)	0.4
STS-Risk-Score (Q1; Q3)	6.7% (4.2-12.8)	8.5% (5.1; 15.3)	0.2	6.4% (4.2; 12.8)	8.5% (5.1; 15.3)	0.06
NYHA class I	0.5%	0%	1	0.7%	0%	1
NYHA class II	5%	4%		3.9%	4%	
NYHA class III	69.6%	70.4%		71.1%	70.4%	
NYHA class IV	24.9%	25.7%		24.3%	25.7%	
COPD	17.7%	23%	0.3	22.4%	23%	1
Coronary artery disease	62.4%	64.5%	0.8	64.5%	64.5%	1
Prior CABG surgery	22.6%	25%	0.6	31.6%	25%	0.1
Prior PCI	55.2%	48.7%	0.3	55.9%	48.7%	0.4
Diabetes mellitus	30.3%	36.2%	0.2	30.9%	36.2%	0.3
Art. Hypertension	79.2%	81.6%	0.8	76.3%	81.6%	0.3
Prior Stroke	10.4%	11.2%	0.8	9.9%	11.2%	0.9
Pre-existing ICD	28.1%	25.7%	0.8	34.9%	25.7%	0.1
Pre-existing CRT	13.6%	9.2%	0.2	16.4%	9.2%	0.06
GFR >60 mL/Min	21.7%	27.0%	0.4	23%	27.0%	0.7
GFR 30-59 mL/Min	59.3%	52.6%		57.2%	52.6%	
GFR <30 mL/Min	18.6%	20.4%		19.7%	20.4%	
NT-pro BNP (ng/L) (Q1; Q3)	2951 (1231; 5521)	2921 (1248; 5938)	0.6	3000 (933; 5771)	2921 (1248; 5935)	0.3
LV function > 45%	40.3%	44.1%	0.8	32.9%	44.1%	0.1
LV function 30-44%	33%	33.6%		35.5%	33.6%	

LV function < 30%	26.7%	22.3%		31.6%	22.3%	
TR grade III	15.8%	29.6%	<0.001	13.8%	29.6%	<0.001
Degenerative MR etiology	28.5%	26.3%		20.4%	26.3%	
Functional MR etiology	63.4%	65.8%	0.8	70.4%	65.8%	0.4
Combined MR etiology	8.1%	7.9%		9.2%	7.9%	
**Procedural characteristics**						
Mean procedure duration (min)	105 ± 60	117 ± 75	0.1	112 ± 73	115 ± 73	0.7
Postinterventional no MR	27.1%	22.4%		27.6%	22.4%	
Postinterventional MR grade I	62.3%	62.5%	0.2	64.5%	62.5%	0.1
Postinterventional MR grade II	9.5%	14.5%		7.9%	14.5%	
Postinterventional MR grade III	0%	0.7%		0%	0.7%	
Length of hospital stay (days) (Q1; Q3)	7 (5; 10)	7 (5; 11)	0.3	7 (5; 10)	7 (5; 11)	0.7
MACCE	5.4% (12)	2%	0.053	5.3%	2%	0.1
In-hospital death from any cause	4.5%	3.3%	0.4	3.3%	3.3%	1
**Heart Failure and anti-arrhythmic medication**						
ACE-/AT1 Inhibitors	76.2%	72.4%	0.6	73%	72.4%	0.8
ARN Inhibitor	7.7%	8.6%	0.7	9.9%	8.6%	0.8
Beta Blockers	88.7%	87.5%	0.2	89.5%	87.5%	0.7
Loop diuretics	90.1%	90.8%	0.9	88.8%	90.8%	0.7
Thiazid diuretics	18.1%	25.7%	0.2	17.1%	25.7%	0.1
Aldosteron antagonists	48.9%	47.4%	0.9	51.3%	47.4%	0.6
Ivabradin	0.9%	0%	0.2	1.3%	0%	0.5
Digitalis	5.4%	17.8%	<0.001	4.6%	17.8%	<0.001
**Oral Anticoagulation**	89.5%	92.3%	0.3	89.5%	92.8%	0.3

Data presented as percentages, mean ± SD or median with first quartile and third quartile 3 (Q1; Q3). AF—atrial fibrillation. NYHA—New York Heart Association. COPD—chronic obstructive pulmonary disease. CABG—coronary artery bypass graft surgery. PCI—percutaneous coronary intervention. ICD—implantable cardioverter defibrillator. CRT—cardiac resynchronization therapy. GFR—glomerular filtration rate. NT-proBNP—N-terminal-pro hormone brain natriuretic peptide. LV function – left ventricular function. TR—tricuspid regurgitation. MR—mitral regurgitation. MACCE—major adverse cardiac and cerebrovascular events. ACE—angiotensin converting enzyme. AT1—angiotensin II type 1 receptor. ARN—angiotensin receptor neprylisin.

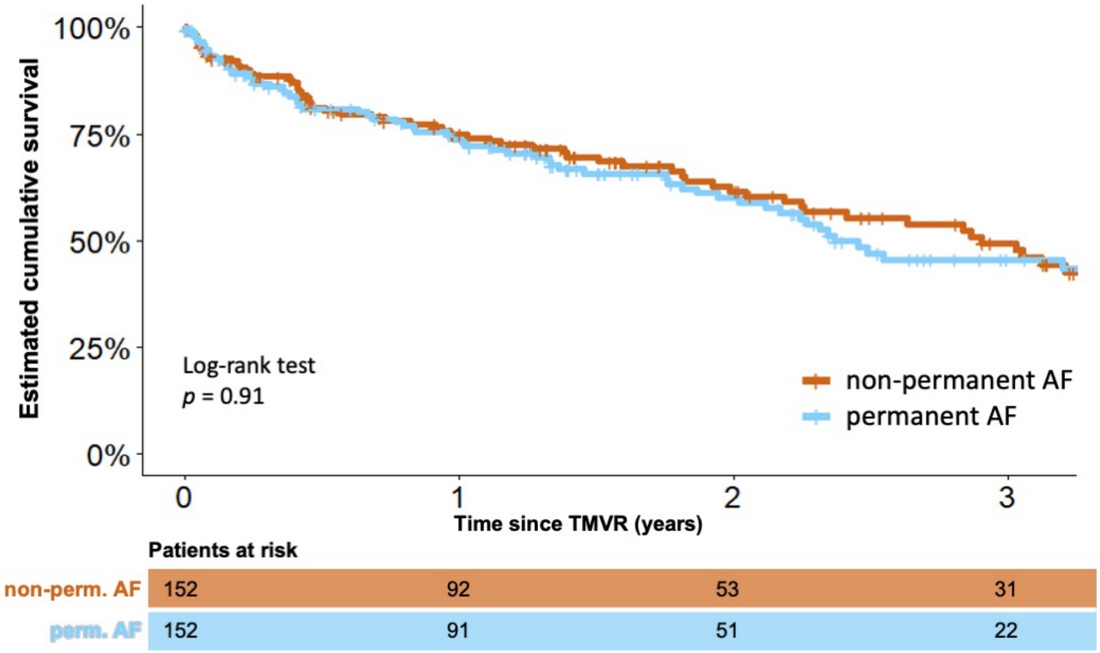

Figure S1. Estimated cumulative survival of patients with permanent and non-permanent atrial fibrillation (AF). Kaplan-Meier plots of patients with permanent and non-permanent AF after propensity score matching (PSM). TMVR—transcatheter mitral valve repair.

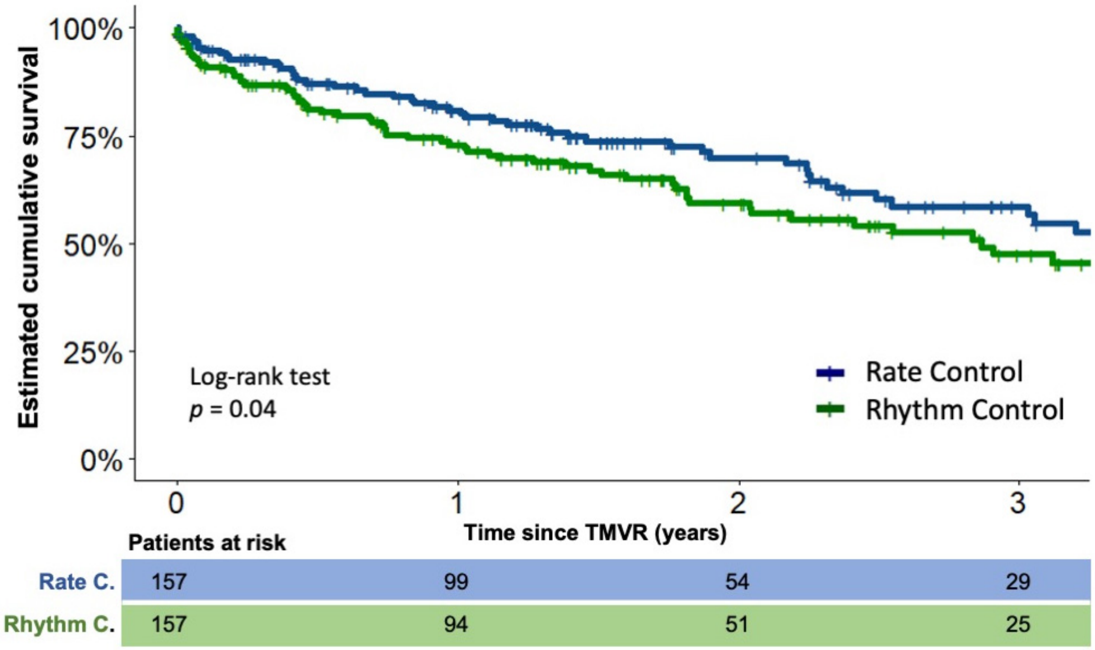

Figure S2. Estimated cumulative survival of patients under rate and rhythm control therapy after exclusion of patients with concomitant ventricular tachycardia. Kaplan-Meier plots of patients under rate and under rhythm control therapy after exclusion of patients with concomitant ventricular tachycardia and after propensity score matching (PSM). TMVR—transcatheter mitral valve repair.
